# Systemic Response to Infection Induces Long-Term Cognitive Decline: Neuroinflammation and Oxidative Stress as Therapeutical Targets

**DOI:** 10.3389/fnins.2021.742158

**Published:** 2022-02-18

**Authors:** Patricia Alves Reis, Hugo Caire Castro-Faria-Neto

**Affiliations:** ^1^Laboratory of Immunopharmacology, Oswaldo Cruz Institute, Fiocruz, Rio de Janeiro, Brazil; ^2^Biochemistry Department, Roberto Alcântara Gomes Biology Institute, Rio de Janeiro State University, Rio de Janeiro, Brazil

**Keywords:** infectious diseases, neuroinflammation, oxidative stress, cognitive impairment, additive therapy

## Abstract

In response to pathogens or damage signs, the immune system is activated in order to eliminate the noxious stimuli. The inflammatory response to infectious diseases induces systemic events, including cytokine storm phenomenon, vascular dysfunction, and coagulopathy, that can lead to multiple-organ dysfunction. The central nervous system (CNS) is one of the major organs affected, and symptoms such as sickness behavior (depression and fever, among others), or even delirium, can be observed due to activation of endothelial and glial cells, leading to neuroinflammation. Several reports have been shown that, due to CNS alterations caused by neuroinflammation, some sequels can be developed in special cognitive decline. There is still no any treatment to avoid cognitive impairment, especially those developed due to systemic infectious diseases, but preclinical and clinical trials have pointed out controlling neuroinflammatory events to avoid the development of this sequel. In this minireview, we point to the possible mechanisms that triggers long-term cognitive decline, proposing the acute neuroinflammatory events as a potential therapeutical target to treat this sequel that has been associated to several infectious diseases, such as malaria, sepsis, and, more recently, the new SARS-Cov2 infection.

## Introduction

It was only at the end of the twentieth century that the concept of the role of glial cells (astrocytes, microglia, and oligodendrocytes) as supporting cells to neurons started to change, and they started to be considered as essential cells to neurological function, as well as players in both physiological and pathological conditions of the central nervous system (CNS) (Bentivoglio et al., 2011; Lana et al., 2021).

Glial cell population can be subdivided into four major groups: microglia, astrocytes, oligodendrocytes, and their progenitors NG2, also known as polydendrocytes (Jäkel and Dimou, 2017). Astrocytes, microglial cells, and oligodendrocytes have several roles on the manutention of brain tissue homeostasis acting in brain physiology, metabolism, and development (Herculano-Houzel, 2014; Jäkel and Dimou, 2017).

Astrocytes are the most abundant glial cells in the CNS having several functions in brain tissue, including the maintenance of water and ion homeostasis, the participation in the tripartite synapse, and contribution to the blood–brain barrier (BBB) (composed of brain microvascular endothelial cells, astrocytes, pericytes, and microglial cells connected by tight junctions) (Persidsky et al., 2006) integrity maintenance. Microglia are the immunocompetent and phagocytic cells of the nervous system, while oligodendrocytes are responsible for synthesis of myelin sheaths that speed up nerve impulse conduction besides providing metabolic support to axons (Kurosinski et al., 2002; Jäkel and Dimou, 2017; Allen and Lyons, 2018).

Both astrocytes and microglial cells have a role in formation of neurovascular unity (NVU), a complex multi-hetero-cellular structure of endothelial cells, neurons, smooth muscle cells, pericytes, and extracellular matrix. Those regulate blood flow and metabolism allowing the controlled exchange of nutrients and metabolic products (Persidsky et al., 2006; Takata et al., 2021). Besides, together with BBB, the blood–cerebrospinal fluid barrier (BCSFB) work in to maintain networks requiring stable extracellular fluid and restore homeostatic balance following CNS insults (Johanson and Johanson, 2018).

Interestingly, NG2 cells or polydendrocytes reside in the corpus callosum or in the subventricular zone, are usually understood as progenitor cells that proliferate around lesions and differentiate into oligodendrocytes, and have also been described with the capacity of converting into neurons and astrocytes and interacting glial with astrocytes and microglia (Nishiyama et al., 2009; Du et al., 2020). In addition, NG2 cells are capable of interact with neuronal cells in the developing and mature CNS. The release of TGF-β by NG2 has been related to the reduction of microglial activation by regulation of CX3CR1–CX3CL1 axis in the microglia–neuron crosstalk, reducing neuroinflammation and neuron toxicity (Zhang et al., 2019). With that in mind, NG2 cells can be proposed as a target to regulate neuroinflammatory conditions in major neurodegenerative diseases, including Alzheimer’s disease, Parkinson’s disease, amyotrophic lateral sclerosis (ALS), and multiple sclerosis (later stages of the disease) (Ransohoff, 2016).

In spite of the brain being considered an “immune privileged” tissue, meaning capable to tolerate the introduction of antigen without eliciting an inflammatory immune response, immune response in the brain can be triggered in several different conditions such as neurodegenerative diseases (for example Parkinson, Alzheimer, and others) and systemic inflammation associated to infectious diseases. Neuroinflammatory events lead to behavioral alterations usually described as sickness behavior (weakness, malaise, listlessness, inability to concentrate, depression, lethargy, and reduction of food intake among other symptoms) (Kelley et al., 2003), Here, we exploit the long-term cognitive sequels after the acute disease, which could be reversible or irreversible, depending on the mechanism of pathogen–host interaction.

## Neuroinflammation

Several stimuli can alter the homeostasis of brain tissue, resulting in alterations in BBB and activation of microglial cells and astrocytes. Microglial cells have been reported as a major player specially in neurodegenerative diseases. Microglial activation has been classically related with the development of M1 proinflammatory phenotype, including shape alteration from resting to activated-ameboid state, with microglia-associated processes, release of cytokines/chemokines, reactive oxygen species, phagocytosis, and elimination of functional synapses (Kreutzberg, 1996; Kurosinski et al., 2002). Cytokine release, mainly IL-1 and TNF-α, triggers astrocyte A1 phenotype, with cytoskeleton size increase, process extension, and expression of glial fibrillary acidic protein (GFAP). This is followed by glial scar formation and the production of proteins of the complement cascade.

Activation of glial cells impacts the BBB/BCSFB integrity, allowing trafficking of immune cells and/or plasma proteins into the brain. Several immune or non-immune events could distort extracellular fluid biochemistry, leading to disabled neuronal/synaptic functions, including alterations in barrier physiology with consequent development of pathophysiology (Johanson and Johanson, 2018). Consequently, NVU is abrogated, with dysregulation of neurovascular coupling, neuron death, gliosis, microglia activation, mural cell transmigration, and BBB breakdown, with consequent increased vascular leakage, transcellular transport, immune cell infiltration, and reduction of tight junctions (Kugler et al., 2021; Shaheryar et al., 2021). In addition, dysregulation of endothelial function by activation of endothelin-1 and nitric oxide (produced by eNOS) induces endothelial activation; cellular inflammatory response; blood flow occlusion; and further neuronal damage, ROS production, and cytokine and matrix metalloprotein-2 and 9 release, which digest proteins responsible for maintaining tight junctions between endothelial cells, compromising the BBB integrity (Tasaki et al., 2014; Park et al., 2018). However, it remains unclear whether BBB dysfunction is a cause or a result of the neuroinflammation.

Neuroinflammation is widely observed in neurodegenerative diseases including Alzheimer’s disease (AD), Parkinson’s disease (PD), multiple sclerosis (MS), and ALS, leading to neuronal dysfunction. Activation of glial cells by pathogen or damage signals induces neurovascular impairment, impacting on neuronal function that could lead to cognitive decline (Sofroniew, 2009; Orihuela et al., 2016; Liu et al., 2020).

Taken together, activation of glial cells by pathogen or damage signals induces neurovascular impairment, impacting on neuronal function that could lead to cognitive decline (Sofroniew, 2009; Orihuela et al., 2016; Liu et al., 2020).

**FIGURE 1:**
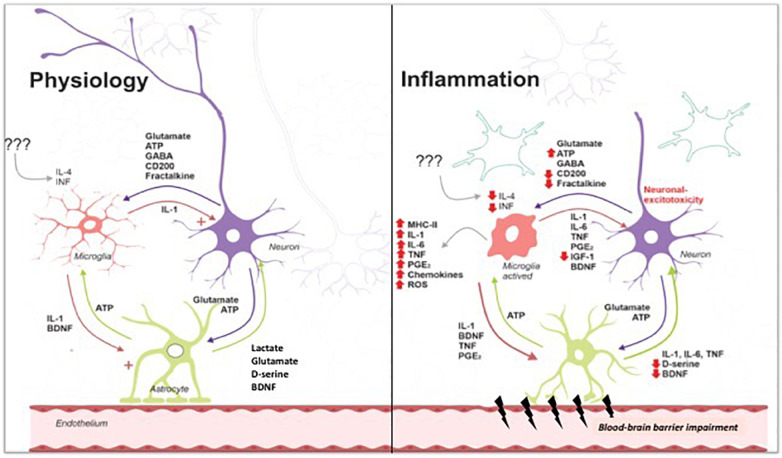
Under physiological conditions, low levels of cytokines contribute to synthesis of proteins to crosstalk among neuron and glial cells. During infectious diseases, inflammation triggers endothelial activation and activation of immune cells. Cytokine/chemokine release leads to activation of blood vessels and blood–brain barrier breakdown. Glial cells (astrocytes and microglial cells) are activated, releasing cytokine/chemokine and reactive oxygen/nitrogen species. Activation of microglia exacerbates cell damage, especially neurons and endothelial cells. Astrocyte activation contributes to cytokines release and blood–brain barrier breakdown, reducing glutamate intake and leading to its accumulation on synaptic cleft. Excessive glutamate triggers excitotoxicity and neuronal death. Tripartite cross talk is lost, contributing to the development of neurological sequelae, in special, cognitive impairment. Image gently provided by Dr. Felipe Dutra.

Figure 1 summarizes the crosstalk between neuron and glial cells: physiologically, cytokines and trophic factors act together to maintain homeostasis, favoring the processes of neurotransmission, including acquisition and memory formation. The systemic inflammatory response activates glial cells, which become immunocompetent, producing inflammatory mediators and contributing to the breakdown of BBB, with consequent neuroinflammation that compromises processes associated with neurotransmission. Together, these alterations, depending on their intensity, trigger subsequent cognitive sequelae that directly impact the quality of life of patients surviving systemic infections.

## Neuroinflammation and Infectious Diseases

Systemic infectious diseases could lead to neuroinflammation (Figure 1), which is defined as a complex inflammatory process in the CNS (Tohidpour et al., 2017).

We and others previously reported the impact of two systemic inflammatory infectious diseases, malaria and bacterial sepsis (Barichello et al., 2006, 2007, 2014; Reis et al., 2010, 2012, 2017), in the brain physiology and in cognition.

Infectious diseases drive immune response by engagement with pattern recognition receptors (PRR). PRRs, such as the Toll-like receptors (TLRs), the nucleotide-binding oligomerization domain (NOD)-leucin rich repeats (LRR)-containing receptors (NLR), the retinoic acid-inducible gene 1 (RIG-1)-like receptors (RLR; RIG-1-like helicases—RLH), and the C-type lectin receptors (CLRs), recognize both pathogen- and damage-associated molecular pattern (PAMP and DAMP, respectively), triggering multiple downstream pathways and transcriptional program activation involved in pathogen clearance. Immune cells (especially macrophages and neutrophils) are recruited, increasing the inflammatory response and tissue damage (Lehnardt, 2010; Iwasaki and Medzhitov, 2015; Andreasson et al., 2016). Most recently, the receptor for advanced glycation end-products (RAGE), multi-ligand receptor of the immunoglobulin superfamily initially characterized and named for its ability to bind to advanced glycation end-products (AGEs), was also described as an able receptor to PAMPs and DAMPs, taking place in inflammatory events, contributing to enhancing tissue damage during neurodegenerative diseases and inflammatory age-related diseases or inflammaging (Teissier and Boulanger, 2019; Wang et al., 2020).

In brain tissue, TNF-α, IL-1β, and IL-6 are specific key players in BBB breakdown and neuronal death. Furthermore, release of ROS and reactive nitrogen (RN) species by glial cells can trigger apoptosis or necrosis in neurons (Bentivoglio et al., 2011; Chitnis and Weiner, 2017; Klein et al., 2017; Tohidpour et al., 2017). In addition, neuronal cells can develop an excitotoxicity process due to excessive glutamate in the synaptic cleft and subsequent extra-synaptic N-methyl-D-aspartate (NMDA) receptor activation that results in a subsequent increase of Ca^+2^ efflux in neuronal cells and the activation of proteins calpain 1 and neuronal nitric oxide. This results in mitochondrial dysfunction and oxidative damage by reactive oxygen and nitrogen species (Fujikawa, 2015). Together, these mechanisms could lead to long-term cognitive decline, which has been shown to be a consequence of several infectious diseases, including sepsis induced by bacterial, viral (example SARS-Cov2), or parasitic infection such as malaria (Iwashyna et al., 2010; Brim et al., 2017; Heneka et al., 2020; Rizzo and Paolisso, 2021).

## Oxidative Stress

Despite the particularities associated with neurodegenerative disorders following infectious or metabolic diseases, oxidative stress seems to be a consensus among then.

Oxidative stress is an imbalance between the production and accumulation of ROS in cells and tissues and the ability of a given biological system to detoxify these reactive products (Pizzino et al., 2017). Cells produce free radicals or products of O_2_ oxy-reduction reactions such as superoxide (O_2_^•⁣–^), hydrogen peroxide (H_2_O_2_), and hydroxyl radical (OH•), under physiological and pathological conditions through mitochondrial electron transport chain, xanthine oxidase, and NADPH–oxidase system (Halliwell and Gutteridge, 2015). Under pathological conditions, ROS can lead to modifications of several biomolecules impacting cell function and to cell death in certain conditions. In addition, many proteins and enzyme have iron in its structure that is capable of generating reactive free radicals through the Fenton reaction, releasing hydroxyl radical and contributing to a cell pro-oxidant status (Meneghini, 1997).

In response to pathogens, macrophage cell lines, including microglia, produce superoxide radical and nitric oxide (by inducible nitric oxide synthase enzyme), resulting in peroxynitrite production that is important to kill invasive microorganisms. However, spilling over peroxynitrite can contribute to nitrosylation of biomolecules, which may result in cytotoxicity (Prolo et al., 2014; Radi, 2018).

The brain has lower levels of antioxidant defense when compared to other organs and, therefore, is more susceptible to oxidative damage (Halliwell, 2006; Bozza et al., 2013). We and others have shown that systemic inflammation, including malaria and sepsis, induce brain oxidative stress and a further reduction of antioxidant defense (Barichello et al., 2006, 2007; Reis et al., 2010). Treatment with antioxidants N-acetylcysteine and desferoxamine reduced cognitive decline associated with both cerebral malaria and sepsis. Inhibition of superoxide delivered from the NADPH oxidase system also prevented oxidative damage, glial activation, and the development cognitive decline (Hernandes et al., 2014). In addition, treatment with lovastatin during experimental cerebral malaria, or simvastatin or atorvastatin in sepsis murine models (Reis et al., 2010, 2012, 2017), also prevented oxidative stress and cognitive decline. Taken together, these evidence point to a central role of oxidative stress in neuronal damage with consequent development of cognitive decline and point to antioxidant agents as potential therapeutic interventions to neurological sequels following systemic infections.

Recently, ferroptosis was identified as a new non-apoptotic, iron-dependent, oxidative mechanism of cell death, which can be caused by transition metal iron and ROS incorporation in polyunsaturated fatty acids into cellular membranes (Dixon et al., 2012; Yang and Stockwell, 2016; Stockwell et al., 2017). Micronutrients, such as selenium and cystine, also have a role on ferroptosis susceptibility, once they are required for the synthesis of glutathione peroxidase 4 (GPX4), responsible for detoxification of free radicals (Jiang et al., 2021). Failure on GPX4 activity enhances lipid peroxidation and thiol system depletion with consequent cell death (Cao and Dixon, 2016; Lei et al., 2019).

Ferroptosis death mechanism has been associated to several neurological diseases, as revised by Ren et al. (2020). To our knowledge, both malaria and sepsis bear not only reduced levels of free thiol but also enhanced lipid peroxidation (Reis et al., 2010, 2017). Although additional investigations are warranted, we believe that ferroptosis could be associated with neuronal death in both cerebral malaria and sepsis, and inhibition of ferroptosis could be a useful therapeutic strategy for these conditions.

## Cognitive Decline

Memory is the ability to retain new information for future use. Basic functions of life require the mechanism of memory formation and consolidation, which are critical for recognizing dangerous signs and social behavior.

Several infectious diseases have been associated to memory impairments: sepsis (mainly in the elderly), acquired immunodeficiency syndrome (AIDS), pneumonia, and cerebral malaria (in this case, mainly young children) (John et al., 2008, 2015; Iwashyna et al., 2010; Bentivoglio et al., 2011; Bangirana et al., 2014; Barichello et al., 2014; Brim et al., 2017). Even the recent coronavirus disease 2019 (COVID-19), caused by the severe acute respiratory syndrome coronavirus-2 (SARS-CoV-2), showed neurologic symptoms, including headache, altered mental status, and anosmia (Rodriguez et al., 2020; Solomon et al., 2020). Psychological alterations such as anxiety, depression, and stress, as well as cognitive dysfunction, have also been described (Pérez-Cano et al., 2020; Rodriguez et al., 2020). The mechanisms of cognitive dysfunction are still not clear, and there is no therapy available to control, prevent, or cure disruption of memory in systemic infections.

One of the brain regions most important to memory formation and consolidation is the hippocampus, the area where the acquisition and “cellular” consolidation of memory take place (Izquierdo and Medina, 1997; Wang and Morris, 2010). These processes take minutes to hours and involve posttranslational modification of synaptic proteins, activation of transcription factors, modulation of gene expression at synapses and cell bodies, and reorganization of pre- and postsynaptic proteins. The end result is synaptic remodeling that makes the memory trace stable (McGaugh, 2000; Nadel and Land, 2000; Lehnardt, 2010; Nadel et al., 2012).

Long-term potentiation (LTP) is associated with memory formation, started by neuronal impulse, whereas NMDA-type glutamate receptors are activated by glutamate, as a result of postsynaptic depolarization through AMPA (α-amino-3-hydroxy-5-methyl-4-isoxazolepropionic acid) receptors and the binding of glutamate. Calcium enters the postsynaptic neuron and activates calcium calmodulin-dependent kinase II (CaMKII), protein kinase C (PKC), and calcineurin. This process is believed to be fundamental to short-term memory (Abel and Lattal, 2001). In sequence, in long-term memory, adenylyl cyclase is activated by Ca^2+^ or by other modulatory inputs, which stimulate adenylyl cyclase through G-protein-coupled receptors. This leads to increases in adenosine 3′,5′-monophosphate (cAMP) levels activating protein kinase A (PKA) that translocate into the nucleus where it phosphorylates the transcription factors cAMP-response element-binding protein (CREB). Other protein kinases, such as CaMKII, CaMKIV, and mitogen-activated protein kinase (MAP-kinase), also regulate gene expression, showing that there is extensive crosstalk among different kinase pathways. These events lead to the production of several proteins, including brain-derived neurotrophic factor (BDNF), which is closely associated with memory consolidation (Weng et al., 1999; Abel and Lattal, 2001; Bekinschtein et al., 2008a).

Interestingly, depending on inflammatory insult, the long-term cognitive sequel could vary. In the experimental model of sepsis, cognitive decline was observed until 30 days post-surgery in survival animals, but it was absent at 60 days post-surgery (Barichello et al., 2005; Tuon et al., 2008b). Conversely, mice that survived cerebral malaria, likewise in patients (John et al., 2008; Bangirana et al., 2014; Christensen and Eslick, 2015; Idro et al., 2016; Brim et al., 2017), presented persistent cognitive impairment that was still present after 90 days (our unpublished observations). Cerebral malaria is characterized by severe inflammatory events in the brain, with an intense vascular activation and intense Th1 response that lead to TCD8^+^-induced endothelial cell death, hemorrhage, oxidative stress, and neuronal death that could lead to irreversible damage and persistent cognitive impairment (Wiese et al., 2006; Dorovini-Zis et al., 2011; Milner et al., 2014; Nacer et al., 2014).

Several mechanisms could be associated with long-term cognitive decline in systemic infectious diseases. So far, we evaluated neuroinflammation, oxidative stress and neuronal death as major events to the development of cognitive sequel. However, it is not established yet which mechanism or combination of several ones triggers cognitive decline due to systemic inflammatory diseases.

Several mechanisms could be associated with long-term cognitive decline in systemic infectious diseases. So far, we evaluated neuroinflammation, oxidative stress, and neuronal death as major events to the development of cognitive sequel. To date, experimental models have given some clues of mechanisms associated to cognitive decline due to infectious diseases. Most of the alterations were observed in the acute phase of infection associated with an intense immune response. Moraes et al. (2015) hypothesize that synaptic dysfunction could be related to reversible cognitive decline due to sepsis by *in vitro* observation of reduction of synaptophysin/PSD-95 rate associated with release of cytokine, especially IL-1β, accomplished through glial activation. In our models of sepsis and malaria, endothelial activation and increased rolling and adhesion of leukocytes, besides blood flow, functional capillary density and cholinergic vasodilator response impairment, resulting on brain edema, release of cytokine/chemokine, and oxidative stress were observed (Reis et al., 2012, 2017). Dysfunctional glutamate metabolism was also observed in both malaria and sepsis, and blocking the NMDA pathway has been proposed (Miranda et al., 2010; Gordon et al., 2015; de Miranda et al., 2017; Juhász et al., 2020).

BDNF, aforementioned to be responsible for plastic changes related to learning and memory, can be a key molecule on the development of cognitive decline understanding. It is well-known that BDNF is synthesized as the precursor pro-BDNF and undergoes cleavage to produce a mature BDNF protein (Lessmann et al., 2003; Bekinschtein et al., 2008b). Curiously, BDNF and pro-BDNF are associated with opposing effects on cellular function; while pro-BDNF preferentially binds p75 NTR receptor, takes place at long-term depression (LTD, associated with weakening spine complexity), and induces apoptosis, mature BDNF form binds to tyrosine kinase receptors (TrkB), promoting cell survival, and facilitates LTP (associated with increasing spine complexity) (McAllister et al., 2003; Woo et al., 2005; Zagrebelsky et al., 2005; Volosin et al., 2006; Friedman, 2010; Gonçalves-Ribeiro et al., 2019). The complexity of this molecule in infectious diseases Is still not clear, and further investigations must be conducted so as to understand the role of BDNF impairment in recovered patients/mice. So far, BDNF has been found to be reduced in mice recovered from sepsis or malaria infection (Bekinschtein et al., 2008a,b; Comim et al., 2012b; Biff et al., 2013). Due to this and other infectious insults, lower levels of BDNF could be associated to neuronal loss or BDNF pathway impairment. However, it is not established yet which mechanism or combination of several ones triggers cognitive decline due to systemic inflammatory diseases.

Our group and others showed that it is possible to reverse/prevent cognitive decline by inhibiting neuroinflammation and reducing glial activation. The use of statins, for example, prevents cognitive decline in both sepsis and malaria diseases. Treatment with statins reduced both systemic and brain cytokine release, impacting on BBB disruption, endothelial activation and damage, and also inhibiting microglial activation. Short- and long-term memory impairment was prevented/reversed by treatment with lovastatin, atorvastatin, and simvastatin, but the molecular mechanism of statins is yet to be described (Reis et al., 2012, 2017). Perhaps, by inhibition of mevalonate release in cholesterol synthesis pathway the prenylation of several proteins associated with proinflammatory response, such as small GTPase Rho, Ras, and Rac, may be affected, and this could be associated to the anti-inflammatory effect of statins and its impact preventing cognitive decline (Palinski and Tsimikas, 2002; Jain and Ridker, 2005; Weber et al., 2005).

Antioxidants also presented a beneficial effect in experimental models of systemic inflammation and cognitive decline. The use of N-acetylcysteine, desferoxamine, and apocynin as a therapeutical support to specific treatment (antimalarial drugs or antibiotic therapy for malaria and sepsis, respectively) reversed cognitive decline in experimental malaria and sepsis. This was accompanied by diminished release of cytokines, controlled microglia activation, and prevention of neuronal death (Barichello et al., 2007; Reis et al., 2010; Hernandes et al., 2014).

As described elsewhere, glial cells, especially astrocytes, participate maintaining homeostasis in brain tissue and controlling neurotransmitter delivery. During neuroinflammation, the recapture of glutamate is impaired, leading to prolonged exposure to glutamate of neuronal cells, with consequent enhancement of sodium and calcium influx. Both ionotropic-NMDA (mainly NR1/NR2B subtype) and metabotropic glutamate receptors are associated with excitotoxic effect of excessive glutamate on synaptic cleft. This event leads to mitochondrial dysfunction and production of reactive oxygen and nitrogen species due overactivation of neuronal nitric oxide synthase, leading to peroxynitrite release, pro-apoptotic factor delivery, and neuronal death (Dong et al., 2009; Wang and Qin, 2010; Allen, 2014; Armada-Moreira et al., 2020). Excitotoxicity is associated with cognitive decline in several degenerative diseases, and this mechanism could be exploited to prevent neurological sequels associated with systemic inflammatory diseases.

## Drug Strategy—Where Are We

To date, there is no effective therapy to treat cognitive sequel associated with systemic infectious diseases. Anti-inflammatory and antioxidant therapy failed to prevent mortality, but these approaches have never been tested as measures to prevent cognitive damage or reduce its severity in survivors. In addition, in intensive care units, treatment focus remains an appropriate management of the systemic infection, without strategies to cognitive impairment development investigation nor treatment.

One of the challenges to propose an effective therapeutical strategy is the complex mechanism of cognitive decline. Also, many observations are done in rodent experimental model, and the translation to human are somehow frustrating. Additional studies in this field are warranted and may unravel key molecular mechanisms to understand cognitive dysfunction after severe infectious syndromes.

As aforementioned, controlling neuroinflammation, oxidative stress, and neuronal death could prevent cognitive decline associated with systemic infectious diseases. Drugs such as statins have shown conflicting evidence in critically ill patients. Morandi et al. (2014) reported that patients under continuous treatment with statins presented reduced sepsis-associated delirium. However, other studies did not confirm the same findings and showed no effect of treatment with statins on neither delirium nor cognitive decline in intensive care patients (Kruger et al., 2013; Needham et al., 2016). One distinct possibility is that the beneficial effect of statins would only be attained in patients under continuous long-term use of the drug, which might indicate that the benefit of statins on cognitive decline is related to their long-term vascular effects (Page et al., 2014; Schultz et al., 2018).

Collaborating with statins findings, some authors suggest that continuous supplemental nutrition, for example with antioxidants or long-chain polyunsaturated fatty acids, could prevent neurodegenerative diseases such as Alzheimer’s disease, Parkinson’s disease, and dementias (revised by Businaro et al., 2021).

In addition to statins, other drugs could be investigated to treat cognitive decline associated with infectious diseases. Several drugs have been developed to treat neurodegenerative diseases and some of this could be tested to cognitive decline associated with infectious diseases as well, based on the analysis of similarity of mechanisms like glial activation, BBB dysfunction, oxidative dysfunction, and neuronal death. Clinical trials with levetiracetam, an antiepileptic drug, were carried out in Alzheimer’s and septic patients with promising results showing inhibition of neuronal dysfunction and inflammatory parameters (Erbaş et al., 2013; Vossel et al., 2021). Memantine, an NMDA antagonist already used to treat Alzheimer patients due to its activity reducing excitotoxic neuronal death (Czarnecka et al., 2021), could also be a candidate to act as an additive therapeutical drug to prevent cognitive sequel associated with infectious diseases. Of course, the safety profile of such drugs must be taken into consideration, especially their combined use with standard therapy in critically ill.

In pre-clinical models, several studies have been conducted in order to control oxidative damage associated to neuroinflammatory response due to systemic infectious diseases. Drugs such as N-acetylcysteine, desferoxamine, and apocynin (inhibitor of NADPH-oxidase system) have already been tested and showed prevention of cognitive decline (Barichello et al., 2007; Reis et al., 2010; Hernandes et al., 2014), but so far, no clinical trials have been developed to test this possibility. Also, erythropoietin, a glycoprotein hormone member of cytokine I family, was tested in preclinical approaches of sepsis and malaria (Wiese et al., 2008; Comim et al., 2012a; Wang et al., 2013). Despite erythropoietin presenting anti-apoptotic, anti-inflammatory, antioxidant, and cytoprotective effects and showing to be beneficial to neurological damage associated to malaria and sepsis, no further clinical use was described so far.

Immune modulators (e.g., rosiglitazone) have already been shown to prevent neuroinflammatory events commonly associated to systemic inflammation. Rosiglitazone is an agonist of peroxisome proliferator-activated receptor gamma (PPARγ), a member of the family of nuclear hormone receptors that function as ligand-activated transcription factors, and is reported to have anti-inflammatory, anti-oxidant, and also neuroprotective properties (Berger and Moller, 2002; Lehrke and Lazar, 2005; Kapadia et al., 2008) and prevented mortality, inflammatory response, neuroinflammation, and vascular dysfunction associated to experimental models of malaria and sepsis (Serghides et al., 2009, 2014; Araújo et al., 2012). The same result was observed in a randomized double-blind placebo controlled trial in Thai patients with *Plasmodium falciparum* malaria (Serghides et al., 2014).

Dexamethasone, a steroidal anti-inflammatory drug, was used in preclinical ligature and cecal puncture model in rats and normalized the adrenal gland weight and plasma levels of corticosterone and adrenocorticotropin hormone, preventing cognitive decline and also depressive-like behavior, showing that reducing inflammatory response has a beneficial effect on CNS function during infectious diseases (Tuon et al., 2008a; Comim et al., 2009). No consolidated data for dexamethasone preventing cognitive decline is available so far.

Other strategies already used was the treatment with antidepressant drugs. Due to its ability to increase neurogenesis and peripheral BDNF delivery in patients and then ameliorate cognitive impairment, those drugs were used in both malaria and sepsis. Antidepressants ameliorate neurological impairment due to sepsis, while lithium presented a beneficial effect on experimental cerebral malaria (Tuon et al., 2007; Dai et al., 2012; Anderson et al., 2016).

Control of inflammation could prevent cognitive decline due to systemic infectious diseases. Despite that the inhibition of cytokine release and other immune pathways seems reasonable, immune response is also indispensable to pathogen clearance, and immunomodulation should be carefully considered.

Antibiotics have been studied to prevent cognitive decline in experimental models of infectious diseases. Beta-lactam antibiotics and tetracyclines have been claimed as neuroprotective drugs due to the ligand and signaling activities beyond their antimicrobial use. For example, ceftriaxone—a beta-lactam antibiotic—increases glutamate transporter-1 expression in neurons and excitatory amino acid transporter-2 in astrocytes, and removal of glutamate ameliorates glutamate excitotoxicity in several *in vivo* and *in vitro* models. Antibiotics ameliorating glutamate excitotoxicity have the potential to prevent a wide spectrum of neurodegenerative diseases and could, therefore, be considered candidates to prevent cognitive decline in severe systemic infectious diseases (Lee et al., 2008; Aminov, 2013; Tikhonova et al., 2017).

Minocycline is a semi-synthetic antibiotic of the tetracycline family and has the capacity to pass the BBB (Aronson, 1980; Jordan et al., 2007; Li et al., 2013). In addition to its microbicidal activity, minocycline has been described with anti-inflammatory properties in neurodegenerative disease models, such as ALS, Alzheimer, Parkinson, and Huntington (Blum et al., 1984; Noble et al., 2009; Kobayashi et al., 2013; Li et al., 2013). Minocycline is known as an inhibitor of microglial activation and to reduce proinflammatory mediators (Kim and Suh, 2009; Plane et al., 2010; Li et al., 2013). Accordingly, Kast (2008) and Apoorv and Babu (2017) showed that minocycline is effective in preventing neurodegeneration and mortality in experimental cerebral malaria. Minocycline was also shown to diminish M1 activation profile during experimental sepsis (Michels et al., 2020). Further investigation must be carried out to confirm the potential of minocycline on the prevention of neuroinflammatory events and cognitive decline.

Here, we provided few considerations of drugs that can be used to manage cognitive decline. However, we are aware that other mechanisms, pathways, or targets also must be relevant. For example, we wonder if the crescent studies of immunometabolism could also bring some clues of mechanisms that trigger organic dysfunction associated with infectious diseases. The understanding of metabolic adaptations during infection at the organismal level may be used to purpose innovative therapeutical strategies that will impact not only on host–pathogen interaction but also controlling the development of neurological sequel (Ayres, 2019; Lercher et al., 2020).

## Concluding Remarks

Despite the growing interest in infection-associated cognitive sequel in recent years, the detailed mechanism of the persistent long-term cognitive decline is still unclear. Data from animal models provided some clues about the general mechanisms involved, but many details need further investigation. In the first place, is not well defined how systemic inflammation is associated with neuroinflammation and cognitive decline. Local brain infection can trigger inflammation, tissue damage, and probably neuronal dysfunction and death, as observed in experimental and human cerebral malaria (Wiese et al., 2006; Milner et al., 2014). Some viruses (Zika, SARS-CoV19, cytomegalovirus, and rubella) present tropism to neuronal cells, inducing dysfunction and death (Carson et al., 2006; Ferreira et al., 2017; Lebov et al., 2018; Pérez-Cano et al., 2020; Rodriguez et al., 2020; Solomon et al., 2020). Intense Th1 immune response seems to induce cognitive decline, mainly memory loss, perhaps due to microvascular alterations, with endothelial activation, adhesion of leukocytes, and capillary rarefaction, which induces hypoxia and metabolic/energetic dysfunction. In addition, in some conditions, immune cells can also infiltrate brain tissue, leading to intensive neuroinflammation and tissue damage, including neuronal death (Perry et al., 2007; Dorovini-Zis et al., 2011).

Several other mechanisms could be associated with systemic immune response and brain dysfunction. Vagal and trigeminal nerves respond to cytokine release, in special IL-1 and IL-6 (Maier et al., 1998; Breit et al., 2018; Zanos et al., 2018). In addition, cytokine can be delivered in the brain by non-covered BBB circumventricular organs and the choroid plexus regions (Banks et al., 2002). Furthermore, BBB activation and permeabilization could allow systemic cytokines to access the brain, activate glial cells, and, consequently, induce neuroinflammation (Sonneville et al., 2013; Nacer et al., 2014; Parra et al., 2021). In systemic infection scenarios, such as observed in sepsis, it is not clear which mechanism triggers neuronal dysfunction. Previous report pointed to cytokines, including IL-1β, inducing synaptic dysfunction and, in consequence, diminishing levels of the neurotrophic factor BDNF (Mina et al., 2013; Comim et al., 2014; Moraes et al., 2015). Long-term consequences of infectious diseases in the brain could be directly associated with the intensity of inflammatory response or due to immune response to the presence of the pathogen in the cerebral tissue (Dorovini-Zis et al., 2011; Ferrari and Moreira, 2011; John et al., 2015; Konradt et al., 2016; Bentivoglio et al., 2018; Lebov et al., 2018). Importantly, some studies point to a post-infection depression behavior, as observed in COVID-19 patients. It is important to mention that depression is commonly associated with sickness behavior. However, some recent studies have shown that persistent depression disorder may also occur. Neurotransmitters’ imbalance could be associated with depression and anxiety, but the long-term impact on mental health still needs further investigation (Pérez-Cano et al., 2020; Rodriguez et al., 2020; Solomon et al., 2020).

Finally, one question that remains unanswered is what is the impact of successive systemic infections during life for neurodegenerative diseases? According to Perry et al. (2007), the acute immune response could exacerbate symptoms and drive the progression of neurodegenerative diseases. Therefore, the relevance of pharmacological approaches that control neuroinflammation and cognitive impairment is clear, providing strategies to control neurodegenerative disorders, like Alzheimer’s, Parkinson, and dementia.

Further studies must be carried out to identify other targets for drug investigation. So far, we have more questions than answers about both the mechanism that leads to cognitive decline and the therapeutical approaches to prevent/reverse this sequel. Those are hard studies to conduct in human patients, and we should, in parallel, work to improve and develop experimental models or systems biology approaches that may help to elucidate the intricate relationship between cognitive decline and severe systemic infectious diseases.

## Author Contributions

PR and HC-F-N conceived and wrote the manuscript. Both authors contributed to the article and approved the submitted version.

## Conflict of Interest

The authors declare that the research was conducted in the absence of any commercial or financial relationships that could be construed as a potential conflict of interest.

## Publisher’s Note

All claims expressed in this article are solely those of the authors and do not necessarily represent those of their affiliated organizations, or those of the publisher, the editors and the reviewers. Any product that may be evaluated in this article, or claim that may be made by its manufacturer, is not guaranteed or endorsed by the publisher.
